# Quality improvement in maternal and reproductive health services

**DOI:** 10.1186/s12884-023-06207-y

**Published:** 2024-01-03

**Authors:** Celia Karp, Erika M. Edwards, Hannah Tappis

**Affiliations:** 1 Johns Hopkins Bloomberg School of Public Health, Baltimore, MD USA; 2 College of Engineering and Mathematical Sciences, University of Vermont, Burlington, VT USA; 3 Robert Larner, MD, College of Medicine, University of Vermont, Burlington, VT USA; 4 Vermont Oxford Network, 33 Kilburn Street, 05401 Burlington, VT USA; 5 JHPIEGO, Baltimore, MD USA; 6 Center for Humanitarian Health, Johns Hopkins University, Baltimore, MD USA

## Abstract

As maternal mortality and morbidity rates stagnate or increase worldwide, there is an urgent need to address health system issues that impede access to high-quality care. Learning from efforts to address the value, safety, and effectiveness of reproductive and maternal health care is essential to advancing quality improvement efforts.

## Main text

Quality health care is key to ensuring human rights in health services and promoting positive health for all [1]. The quality of health services can have direct effects on the effectiveness of interventions or treatments, and indirect effects on health outcomes by influencing health-seeking behavior, patient engagement, self‐efficacy, and psychosocial health. As access to health services has grown in many parts of the world, a greater focus on understanding and improving quality can reduce gaps in progress between utilization of health services and health outcomes [2, 3].

Quality health care is safe, effective, and people-centered with services that are safe, timely, equitable, integrated, efficient, and patient- or family-centered [4]. A clinical and structural lens has historically been used to evaluate the quality of health services. Donabedian’s seminal 1966 quality of care framework, outlining a focus on “structure, process, and outcome,” has grounded research and practice centered on quality improvement [5]. Efforts to promote quality in health services center on enhancing the structures in which services are delivered, including the physical, facility environment; availability of essential medicine, supplies, and equipment; and the presence of trained medical staff to deliver life-saving care. Interpersonal aspects of quality, which fall within the “process” domain of Donabedian’s framework and capture interactions between health providers and patients, have received growing attention in recent decades with a shift toward person-centeredness in health services [6–8].

Quality improvement (QI) is a systematic approach, guided by data, to improve health care delivery. QI initiatives can be led by a team in a single clinic or unit, or by quality improvement professionals working across health care systems – although ideally, every single person in a health care setting should be an advocate for quality care. Developing a culture of quality, identifying QI champions, and establishing QI teams that include patients or families are ways to start [9]. As part of their charge, teams can review data, undertake small tests of change, and monitor progress. Such focus, which requires time and a coordinated mindset and effort, can create significant transformations.

### Rationale for this Collection

A focus on ensuring high-quality health services is particularly salient for reproductive and maternal health services. Progress in reducing maternal mortality has stalled. While Africa and Asia shoulder the greatest mortality burden, the most recent UN estimates show stagnation or increases in maternal mortality in nearly all regions of the world – including in Europe, North America, Latin America and the Caribbean [10]. This stagnation has been attributed to reproductive and maternal health slipping down the global political agenda, along with increases in inequities, shortfalls in resources, and efforts of political and religious entities to limit the socioeconomic rights of women.

In all countries, as women^1^ and couples navigate their unique life circumstances, make complex decisions, and engage in behaviors and practices related to sex, pregnancy, and childbearing, the delivery of high-quality, person-centered care is central to supporting them in achieving their reproductive goals. Person-centered care is paramount for sensitive and preference-specific healthcare, including reproductive and maternal care, as it ensures that services are tailored to individuals’ needs and preferences. In the case of reproductive and maternal health, preferences related to contraceptive method attributes (e.g., efficacy, duration, side effects) and childbirth conditions (e.g., location, position, support personnel) play a critical role in shaping individuals’ experiences of care and may impact subsequent care-seeking [11, 12].

The UN report on trends in maternal mortality from 2000 to 2020 urges collective action to address health system issues that impede access to safe, quality, respectful and affordable pregnancy care and sexual and reproductive health and rights for all women and girls [10]. Improving quality of care in maternal and reproductive health requires support by and for providers at the community, facility, district, and broader system and societal levels. Learning from efforts to analyze and address the value, safety, and effectiveness of reproductive and maternal health care in public and private health care systems around the globe is essential to advancing quality improvement efforts.

This Collection was launched in *BMC Pregnancy and Childbirth* and *BMC Women’s Health* to highlight research that investigates ways in which the delivery of high-quality women’s health services can be strengthened to align with clinical standards, evidence-based practices, and individuals’ preferences for care. Facilitators and barriers to quality health service delivery will be explored and learning from recent and ongoing initiatives showcased as a call for collective action to continue investing in reproductive and maternal health care quality improvement.

## Data Availability

Not applicable.

## Footnotes

1 We use the term women throughout this editorial but recognize that people who do not identify as women can become pregnant and require the same considerations in terms of understanding their preferences for care.
